# Age, Gender and Normalization Covariates for Spinal Cord Gray Matter and Total Cross-Sectional Areas at Cervical and Thoracic Levels: A 2D Phase Sensitive Inversion Recovery Imaging Study

**DOI:** 10.1371/journal.pone.0118576

**Published:** 2015-03-17

**Authors:** Nico Papinutto, Regina Schlaeger, Valentina Panara, Alyssa H. Zhu, Eduardo Caverzasi, William A. Stern, Stephen L. Hauser, Roland G. Henry

**Affiliations:** 1 Department of Neurology, University of California San Francisco, San Francisco, California, United States of America; 2 Department of Neurology, University of Basel, Basel, Switzerland; 3 ITAB—Institute of Advanced Biomedical Technologies, University “G. D'Annunzio”, Chieti, Italy; 4 Bioengineering Graduate Group, University of California San Francisco, San Francisco, California, United States of America, and University of California, Berkeley, California, United States of America; 5 Department of Radiology and Biomedical Imaging, University of California San Francisco, San Francisco, California, United States of America; University of Toronto, CANADA

## Abstract

The source of inter-subject variability and the influence of age and gender on morphometric characteristics of the spinal cord, such as the total cross-sectional area (TCA), the gray matter (GM) and white matter (WM) areas, currently remain under investigation. Understanding the effect of covariates such as age, gender, brain volumes, and skull- and vertebra-derived metrics on cervical and thoracic spinal cord TCA and GM areas in healthy subjects would be fundamental for exploring compartment specific changes in neurological diseases affecting the spinal cord. Using Magnetic Resonance Imaging at 3T we investigated 32 healthy subjects using a 2D phase sensitive inversion recovery sequence and we measured TCA, GM and WM areas at 4 cervical and thoracic levels of the spinal cord. We assessed age and gender relationships of cord measures and explored associations between cord measures and a) brain volumes and b) skull- and vertebra-derived metrics. Age and gender had a significant effect on TCA, WM and GM areas (with women and elderly having smaller values than men and younger people respectively), but not on the GM area/TCA ratio. The total intracranial volume and C3 vertebra dimensions showed the highest correlations with cord measures. When used in multi-regression models, they reduced cord areas group variability by approximately a third. Age and gender influences on cord measures and normalization strategies here presented might be of use in the study of compartment specific changes in various neurological diseases affecting the spinal cord.

## Introduction

Spinal cord (SC) atrophy is a common and clinically relevant aspect of various diseases, the effects of which are either confined to the spinal cord or involve the whole central nervous system, such as adrenomyeloneuropathy [1,2], amyotrophic lateral sclerosis [3] and multiple sclerosis (MS) [4,5].

Progressive disability in MS is currently thought to be largely driven by spinal cord involvement that may affect both the gray matter (GM) and white matter (WM) compartments [6,7], impacting clinical presentation and disease course.

Magnetic Resonance Imaging (MRI) recently enabled in-vivo morphometric assessments of the WM and GM areas of the spinal cord, by using T2* weighted imaging [8–11] or phase-sensitive inversion recovery (PSIR) imaging [12–14]. Understanding the sources of inter-subject variability of the measured SC total cross-sectional areas (TCA), GM and WM areas and their relationship with age and gender might improve both sensitivity and specificity of the morphometric assessments in detecting disease-associated changes.

Prior MRI studies that explored age and gender effects on spinal cord metrics in healthy controls were mostly focused on TCA (or cord volume) measurements at the cervical levels [15–24]. In these studies there was strong agreement regarding the influence of sex on cervical TCA or volumes, with men having larger values compared to women. The majority of the cited papers reported a moderate decrease of cervical cord areas with age, but this behavior was not consistently observed in all studies.

These findings are partially in contrast with some very recent data based on T2* imaging on healthy subjects, investigating age and gender effects not only on TCA but also on GM and WM metrics within the spinal cord [11]. The authors did not find any gender influence on most of the investigated metrics (the cross-sectional area, the transverse and anteroposterior diameters, the WM% and the width of the anterior and posterior horns), with the exception of the posterior horn width. Age-related differences were only observed for anteroposterior diameter and white matter percentage, not for any of the other metrics, and were only investigated for the cervical cord. A specific description of the chosen metrics at the different anatomical levels was not reported.

Three of the above cited works [16,17,21] also explored the correlation of brain and/or skull volumes with the upper cervical TCA on healthy subjects to define potential normalization strategies. All these papers reported very strong correlations of the total intracranial volume (TICV) and of brain GM, brain WM and total brain volumes with the cervical TCA.

Finally, several studies aimed at identifying potential normalization strategies for cervical TCA or volume measurements in MS [15–17,25–28] using proportion or residual approaches [29]. The normalization metrics explored in these studies included the TICV, the number of slices/length of the spinal cord, the lumbar enlargement cord area (LECA), the thecal sac volume, the maximum intracranial/skull cross-sectional area, and the body mass index. The effect of normalization strategies was assessed in terms of the prediction of clinical disability and the ability to detect differences among disease phenotypes. However, the results of these studies are partially conflicting and a consensus on which metric best help in reducing the substantial inter-subject variability of TCA is still lacking.

Normalization for LECA noticeably increased the correlation of cervical TCA with clinical disability expressed by the expanded disability status scale (EDSS) on a group of 29 MS patients [15]. This normalization is based on the assumption that the disease doesn’t affect the lower portion of the spinal cord. Normalization of cervical volumes for the number of slices/length of the spinal cord improved the detection of differences between MS phenotypes or MS patients and healthy controls [25,26,30]; this method essentially translates a measurement of volumes into a measurement of a mean cross-sectional area.

The use of TICV seemed to have a limited utility [16,17,25–27,30]. The negligible effect of TICV observed in these papers, despite its strong correlation with TCA, could anyway be due to an interplay between TICV and gender [21], sometimes also used in the corrections, or to the fact that the normalization with TICV was mainly tested on relapsing remitting patients on which initial inflammation can mask cervical TCA atrophy [17,30].

None of these studies provided normalization strategies with respect to the analysis of the GM/WM components of the spinal cord.

The aim of the present work was to extend the previous studies by exploring the effect of covariates such as age and gender on TCA, WM and GM areas at 4 selected cervical and thoracic levels (C2-C3, C3-C4, T8-T9 and T9-T10) of the spinal cord on healthy controls using an acquisition and measurement procedure based on 2D PSIR imaging that was recently shown to allow sensitive and clinically feasible spinal cord assessment of gray and white matter areas in efficient acquisition times of <2 min per level, with high test-retest, intra-rater and inter-rater reliability [13,14].

Moreover, we aimed to investigate at cervical and thoracic levels associations among cord measures and between cord measures and a) brain volumes and b) skull- and vertebra-derived metrics to develop potential normalization strategies to reduce inter-subject variability.

## Materials and Methods

### Imaging protocol

Thirty-two healthy subjects (14 men: mean age 46.8±13.4; 18 women: mean age 50.4±15.1; total cohort: age 28–78; mean 48.8±14.3) underwent MRI scanning on a Siemens 3T Skyra system equipped with a 20-channel head-neck coil and a 32-channel spine coil. To minimize neck movement during the examination, each subject was provided with a MR compatible cervical collar [9].

All subjects had no history of psychiatric, neurological or cognitive impairment and provided written informed consent to participate in this study. Research was performed in compliance to the Code of Ethics of the World Medical Association (Declaration of Helsinki) and the standards established by our Institution. The Committee on Human Research at the University of California, San Francisco (UCSF) approved the study protocol. Written informed consent was obtained from all participants.

A standard high resolution T1 weighted acquisition of the brain (sagittal 3D-MPRAGE, 1 mm^3^ isotropic resolution, acquisition time ∼5:30 min) and 2D PSIR acquisitions with an optimized 2D PSIR protocol (axial in plane resolution 0.78*0.78 mm^2^, slice thickness 5 mm, matrix 256x256, TR/TE/TI = 4000/3.22/400 ms, angle = 10°, 3 averages, acquisition time: 1:50 min, magnitude and phase-sensitive images reconstructed) were acquired for each participant. 2D-PSIRs were acquired at four intervertebral disc levels representative of the cervical and the low thoracic portions of the spinal cord (C2-C3, C3-C4, T8-T9 and T9-T10) in a total acquisition time < 8 minutes. Standard T2 weighted sagittal acquisitions of the whole spinal axis were also acquired to prescribe the PSIRs, using the disc as reference and positioning the slices perpendicular to the spinal cord.

C2-C3 is the standard level explored in MS studies [12,16,17,31–33], while the C3-C4 and the selected thoracic levels, just above the lumbar enlargement, are much less explored in literature. T8-T9 and T9-T10 were chosen since we hypothesize that the GM and total cord areas at these levels might be potential markers of spinal cord function regarding the lower limbs.

### Area and metric measurements

Total spinal cord and GM areas at the different levels were calculated on the phase-sensitive reconstructed images using the software Jim (Version 6.0, Xinapse Systems, Northants, United Kingdom; http://www.xinapse.com). The total cross-sectional area (TCA) estimates were segmented in a semi-automated way [34]; briefly this was done using the cord finder toolkit with fixed settings (nominal cord diameter 8mm, number of shape coefficients 24, order of longitudinal variation 12). The marker requested by the toolkit was positioned on the mid-sagittal WM, directly posterior to the gray commissure.

**Fig 1:**
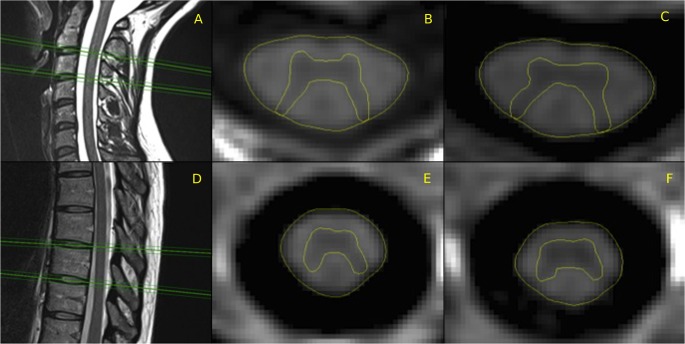
Example of acquisition and segmentation. Example of acquisition and total cross-sectional area (TCA) and gray matter (GM) segmentation on a representative healthy subject. Left: Sagittal images, positioning of 2D PSIR slices (A: cervical portion, D: thoracic portion). Right: segmentation on the phase-sensitive reconstructed images (B: C2-C3, C: C3-C4, E: T8-T9, F: T9-T10 disc levels).

**Fig 2:**
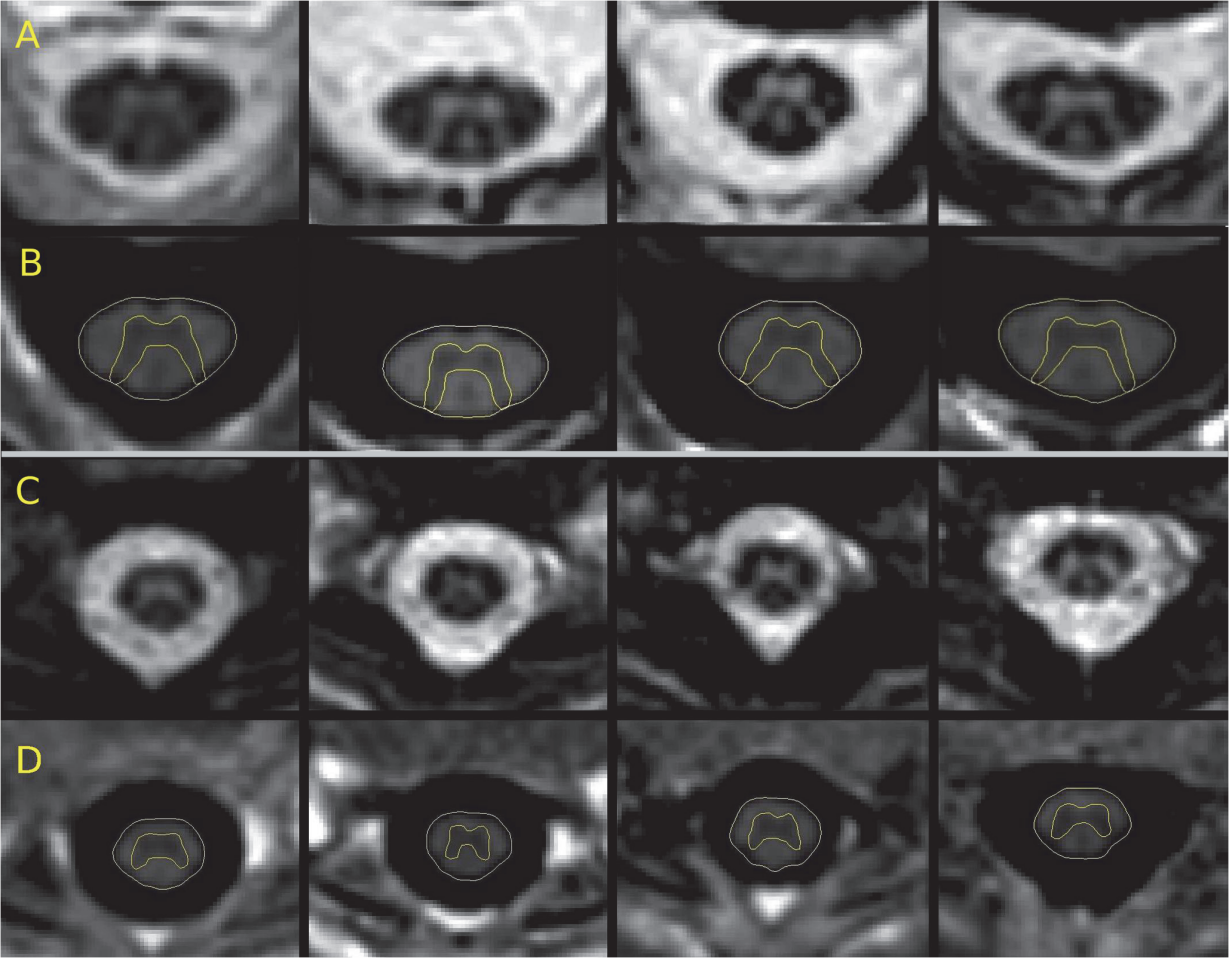
C2-C3 and T9-T10 segmentation examples on 4 healthy subjects. Images acquired for 4 different healthy subjects: A and B: C2-C3 level; C and D: T9-T10 level. In rows A and C the magnitude reconstructions are reported to appreciate the quality of the acquisitions, while in rows B and D the phase-sensitive reconstructed images that were used for both the total cross-sectional area (TCA) and gray matter (GM) segmentations (yellow) are reported. Phase-sensitive reconstructed images allowed good intra- and inter-operator reliability of both the area measurements on a single image.

The resolution of images, which is slightly lower than the resolution used in previous work based on T2* imaging [8,9,11], was chosen in view of later clinical feasibility to minimize the acquisition time. Despite this potential shortcoming, both the inter- and intra-operator reliabilities of the acquisition and measurement procedure were shown to be high [13,14] and non-inferior to reliability assessments in the earlier studies based on a slightly higher resolution. Therefore, a single neuroradiologist with 6 years of experience (VP) manually segmented the GM area three times using Jim, and the average GM area was calculated. The SC WM area was calculated as the difference between the TCA and the average GM area. Examples of acquisition and segmentation are reported in Fig. 1 and Fig. 2.

Segmentation and parcellation of the T1 weighted anatomical structural images was performed with Freesurfer (available for download at http://surfer.nmr.mgh.harvard.edu/) using the Desikan-Kyliany Atlas, in order to obtain the total intracranial volume (TICV), subcortical GM, cortical GM, total WM, thalamus, cerebellar and brainstem volumes [35–37]. On the MPRAGE, resampled to be in axial planes correctly oriented, we measured the area of the foramen magnum. The foramen magnum perimeter was manually delineated on the most inferior slice in which the bone was completely visible.

**Fig 3:**
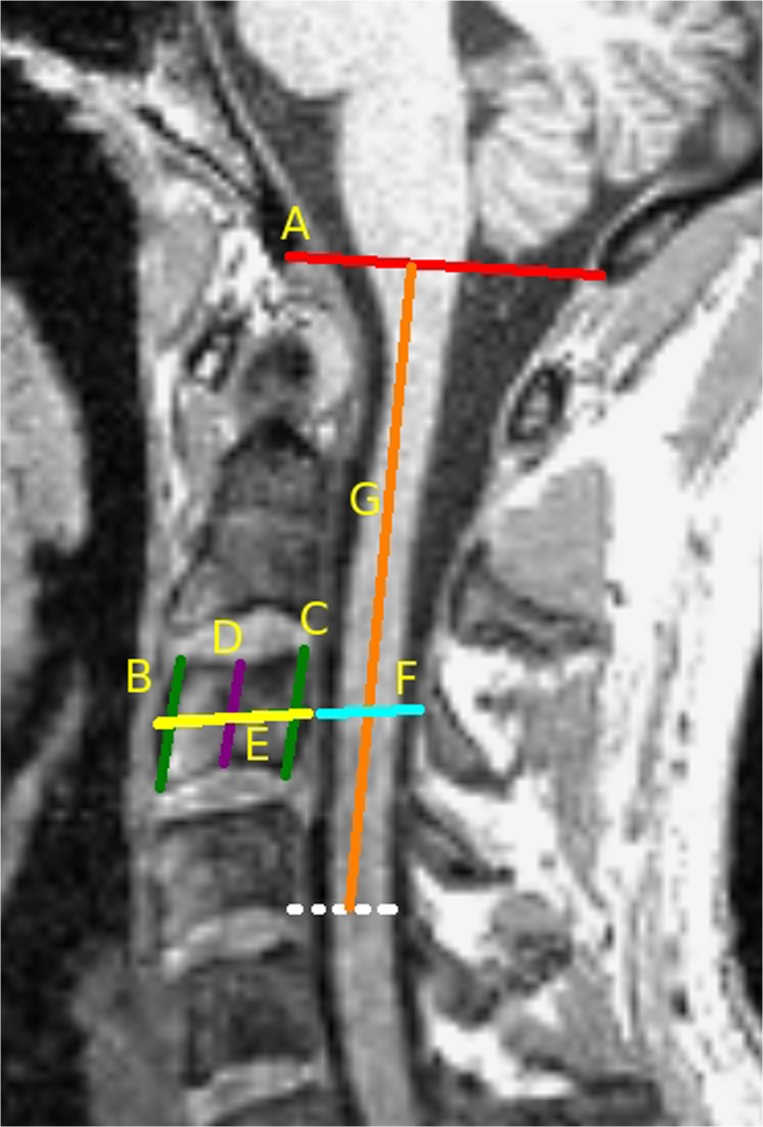
Definition of spinal skeletal based metrics. Cervical skeletal based metrics defined on the sagittal MPRAGE: A: distance between the opisthion and the basion (McRae's line); B: anterior height (AP) of the C3 vertebra; C: posterior height (PH) of the C3 vertebra; D: central height of the C3 vertebra (middle_vertebra_height); E: C3 anterior-posterior vertebral body diameter (ap_vertebra_diameter); F: spinal canal diameter at the C3 vertebra level (ap_canal_diameter); G: Distance between the McRae's line and the bottom of the C4 vertebral body (McRaes_to_C4).

On the sagittal MPRAGE the following 9 skeletal based metrics were defined as illustrated in Fig. 3:

The anterior-posterior vertebral body diameter (*ap_vertebra_diameter*) and spinal canal diameter (*ap_canal_diameter*) were measured at the mid portion of the C3 vertebra [38,39].
Anterior height (AH), posterior height (PH) [40], and central height (*middle_vertebra_height*) [39] of the C3 vertebra were measured.
  From these latter measures, three metrics were derived:
  Average of AH and PH (*mean_vertebra_height*)
*sagittal_vertebra_area* = mean_vertebra_height x ap_vertebra_diameter
  bi-trapezoidal approximation of the vertebra area: *sagittal_area_trapez* = 2 x [(mean_vertebra_height + middle_vertebra_height) / 2 x ap_vertebra_diameter / 2]
Distance between the opisthion and the basion (*McRae's line*) [41]
Distance between the McRae's line and the bottom of the C4 vertebral body (*McRaes_to_C4*)
nasion-inion distance (*nasion-inion*)

### Statistical analysis

Statistical analysis was performed using IBM SPSS Statistics, Version 21, IBM Cooperation, 2012 and JMP Statistics (http://www.jmp.com), Version 11, 2013 SAS Institute. The values for TCA, GM, WM area and GM area/TCA for all the 4 levels, brain volumes and the skull- and vertebra-derived metrics were assessed for normality of the distribution of data using the Shapiro-Wilk W tests.

### Age and gender influences

We assessed differences of PSIR measures between men and women at the C2-C3 and T9-T10 disc levels by calculating least square means with adjustment for age. In the same way, the influence of age on PSIR measures was assessed with gender as a covariate, dividing the total cohort in two groups (taking the median age as a cut-off).

To verify if possible gender differences could be explained by the physical characteristics of the subjects (height and weight) we also explored the effect of age, gender, height and weight on the spinal cord areas at cervical and thoracic levels in a multivariate analysis (taking into account multiple comparison corrections).

### Cord measures associations within the cord and with brain/vertebra metrics

Correlations of TCA and GM area among the four levels were investigated calculating Pearson product-moment correlation coefficients.

The correlations between TCA and GM area at the C2-C3 and T9–10 levels and brain volumes and skull- and vertebra-derived measures (TICV, *nasion-opistion*, foramen magnum area, *McRae's line*, *McRaes_to_C4*, *ap_canal_diameter*, *ap_vertebra_diameter*, *middle_vertebra_height*, *mean_vertebra_height*, *sagittal_vertebra_area*, *sagittal_area_trapez*) were also assessed by Pearson product-moment correlation coefficients.

To narrow down the number of candidate variables for normalization, we focused our further analysis only on those metrics less likely to be altered by neurological diseases that affect both brain and spinal cord, such as skull- and vertebra-derived metrics.

Among these, we selected variables having a correlation coefficient greater than 0.45 with at least one of either TCA or SC GM area. We performed a multi-linear regression analysis with the cord metrics at the C2-C3 disc level as the outcome variables and gender and age as covariates.

We normalized both the TCA and GM area values using a regression-based residual method [17,29] applying the following formula derived from the results of the regression analysis models with an adjusted R^2^ > 0.35:

$${\text{A}}^{\text{i}}{}_{\text{pred},}={\text{A}}^{\text{i}}{}_{\text{meas}}+\text{a}\;\left(\;{\text{X}}_{\text{mean}}-{\text{X}}^{\text{i}}{}_{\text{meas}}\right)+\text{b}\;\left(\;{\text{Y}}_{\text{mean}}-{\text{Y}}^{\text{i}}{}_{\text{meas}}\right)+\text{c}\;\left(\;{\text{Z}}_{\text{mean}}-{\text{Z}}^{\text{i}}{}_{\text{meas}}\right)+\text{d}\;\left(\;{\text{J}}_{\text{mean}}-{\text{J}}^{\text{i}}{}_{\text{meas}}\right)+..$$ Eq.1

where A^i^_meas_ is the measured SC area in a given subject i, A^i^_pred_ is the resulting normalized area, a,b,c,d,… are the regression coefficients derived from the multi-linear fit, X _mean_, Y _mean_, Z _mean_, J _mean_, … are the mean values of the skull/cord metrics of the 32 subjects group and X^i^_meas_, Y^i^_meas_, Z^i^_meas_, J^i^_meas_, … are their measured values in the subject i.

The effect of the normalization by a particular model was evaluated by comparing the relative standard deviations (ratios of the group standard deviation and the respective means, %RSD) between the measured data and the values generated by the respective correction formulas. The best models found at C2-C3 were applied to the other 3 spinal cord levels to normalize TCA and GM areas, and the respective adj. R^2^ and the %RSD were calculated.

## Results

### Area and metric measurements

TICV, subcortical GM, cortical GM, total WM, precentral GM, thalamus, cerebellar and brainstem volumes, *nasion-opistion*, foramen magnum area, *McRae's line*, *McRaes_to_C4*, *ap_canal_diameter*, *ap_vertebra_diameter*, *middle_vertebra_height*, *mean_vertebra_height*, *sagittal_vertebra_area*, *sagittal_area_trapez* were measured on 30 subjects, since the MPRAGE was not acquired in 2 subjects. According to the Shapiro-Wilk W tests all metrics were normally distributed.

The quality of the PSIR images was consistently high in all 32 subjects at all levels of the spinal cord and high GM/WM contrast. For 2 subjects who were scanned at the beginning of the study the 2 thoracic levels were not acquired.

The TCA, GM area (in mm^2^) and GM area/TCA (in %) measured on the group were, respectively (mean ± standard deviation): **C2-C3:** 79.7 (± 7.0), 19.4 (± 1.9), 23.8 (± 2.4); **C3-C4:** 84.1 (± 7.1), 21,9 (± 1.9), 25.4 (± 1.6); **T8-T9:** 44.3 (± 3.1), 10.3 (± 1.6), 23.3 (± 3.0); **T9-T10:** 45.3 (± 3.5), 11.1 (± 1.3), 24.5 (± 2.0).

TCA and GM area values showed strong correlations among the 4 measured levels (Table 1). Particularly strong correlations were evident between adjacent cervical (r = 0.89 and r = 0.58 for TCA and GM areas, respectively) and thoracic (r = 0.89 and r = 0.63 for TCA and GM areas, respectively) levels.

**Table 1:** Cord measures associations within the cord.

		C2-C3		C3-C4		T8-T9		T9-T10	
		TCA	GM	TCA	GM	TCA	GM	TCA	GM
C2-C3	TCA	**1**	**0.65**	**0.89**	**0.76**	**0.74**	**0.55**	**0.65**	**0.63**
			**(<0.001)**	**(<0.001)**	**(<0.001)**	**(<0.001)**	**(0.002)**	**(<0.001)**	**(<0.001)**
	GM		**1**	**0.51**	**0.58**	**0.68**	**0.38**	**0.59**	**0.52**
				**(0.003)**	**(0.001)**	**(<0.001)**	**(0.041)**	**(0.001)**	**(0.004)**
C3-C4	TCA			**1**	**0.85**	**0.63**	**0.55**	**0.57**	**0.62**
					**(<0.001)**	**(<0.001)**	**(0.002)**	**(0.001)**	**(<0.001)**
	GM				**1**	**0.62**	0.30	**0.54**	**0.57**
						**(<0.001)**	(0.104)	**(0.002)**	**(0.001)**
T8-T9	TCA					**1**	**0.49**	**0.89**	**0.76**
							**(0.006)**	**(<0.001)**	**(<0.001)**
	GM						**1**	**0.49**	**0.63**
								**(0.006)**	**(<0.001)**
T9-T10	TCA							**1**	**0.74**
									**(<0.001)**
	GM								**1**
								

Pearson coefficients and respective p values (reported in brackets) for the relationships of total cross-sectional area (TCA) and gray matter (GM) area in the cohort of 32 subjects at the C2-C3, C3-C4, T8-T9 and T9-T10 disc levels. Statistically significant values are reported in bold. Since this was an explorative qualitative analysis, no correction for multiple comparison was performed.

At the 4 selected levels TCA, WM, GM area and GM area/TCA were normally distributed, with the only exception being GM area at the T8-T9 level.

This latter finding, the strong correlations among adjacent levels, and a higher reliability of the GM area measurements guided us to select the C2-C3 level and the T9-T10 level as representative of the cervical and thoracic section of the spinal cord for the further analyses.

### Age and gender influences

Gender differences of PSIR measures at the C2-C3 and T9-T10 disc levels are reported in Table 2. When adjusted for age, women generally had smaller TCA and SC GM areas compared to men. There was also a trend toward smaller SC WM areas in women; however, no statistical significant difference of the SC GM area/TCA ratio was found between sexes.

**Table 2:** Gender influence on spinal cord areas.

	Disc level	Sex	Adj. mean	StdErr (mean)	Mean diff.	StdErr (diff.)	p	95%-Confidence Interval (diff.)	
TCA (mm^2^)	C2-C3	M	82.33	1.62	4.60	2.17	**0.0430**	0.15	9.04
		F	77.73	1.43					
Mean WM	C2-C3	M	62.09	1.42	3.09	1.89	0.1147	−0.79	6.97
area (mm^2^)		F	58.99	1.25					
Mean GM	C2-C3	M	20.24	0.46	1.51	0.62	**0.0220**	0.23	2.78
area (mm^2^)		F	18.73	0.41					
GM area/TCA	C2-C3	M	24.65	0.50	0.52	0.67	0.4452	−0.85	1.89
ratio (%)		F	24.13	0.44					
TCA (mm^2^)	T9-T10	M	47.01	0.73	3.05	0.98	**0.0042**	1.05	5.05
		F	43.96	0.64					
Mean WM	T9-T10	M	35.19	0.64	1.79	0.85	**0.0453**	0.04	3.54
area (mm^2^)		F	33.39	0.56					
Mean GM	T9-T10	M	11.82	0.27	1.26	0.36	**0.0017**	0.52	2.00
area (mm^2^)		F	10.56	0.24					
GM area/TCA	T9-T10	M	25.20	0.54	1.2	0.72	0.1076	−0.28	2.70
ratio (%)		F	23.99	0.47					

PSIR measures at the C2-C3 and T9-T10 disc levels of men and women using linear regression with age as covariate. Mean values are least square means with adjustment for age. Adj.: adjusted; StdErr: standard error, Diff.: difference between means, TCA: total cross-sectional area, GM: gray matter, WM: white matter. p-values are 2-sided.

Weight was always found to be negligible in explaining the variance of cord areas, while height showed to be more important for some tissues and some levels but with no consistency. Regardless, height never reached a p-value smaller than 0.1 in the multivariate analyses at any levels and for any of the metrics.

**Fig 4:**
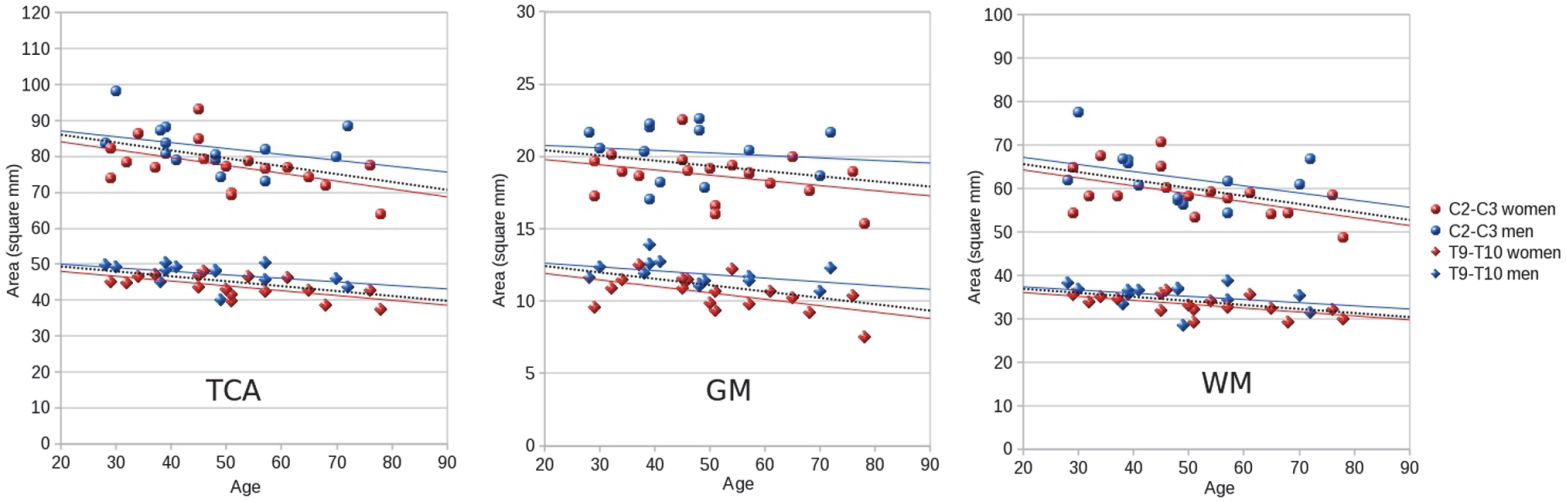
Influence of age on spinal cord areas. Total cross-sectional area (TCA), gray matter (GM) and white matter (WM) areas in function of age at the C2-C3 and T9-T10 disc level for men (blue) and women (red). Areas are reported in mm^2^. Linear regression lines are reported (blue: men; red: women; black dotted: whole cohort -cervical level: n = 32; thoracic level: n = 30−).

TCA, GM and WM cord areas as a function of age are shown in Fig. 4. In general, older subjects had smaller total cord and WM areas and a trend to smaller GM areas compared to younger subjects when taking the gender-adjusted group median as a cut-off (Table 3). The TCA showed an average reduction of about 2 mm^2^ per decade at the C2-C3 level and 1.3 mm^2^ at the T9-T10 level, while the average SC GM reduction was of the order of 0.3–0.4 mm^2^ per decade at both the levels. The ratio between SC GM area and TCA, however, did not show a significant age effect.

**Table 3:** Age influence on spinal cord areas.

	Disc level	Age group	Adj. mean	StdErr (mean)	Mean diff.	StdErr (diff.)	P	95%-Confidence Interval (diff.)	
TCA (mm^2^)	C2-C3	< 48 years	83.58	1.39	7.12	1.98	**0.0012**	3.06	11.19
		≥ 48 years	76.46	1.42					
Mean WM	C2-C3	< 48 years	63.51	1.24	5.96	1.76	**0.0021**	2.36	9.57
area (mm^2^)		≥ 48 years	57.55	1.26					
Mean GM	C2-C3	< 48 years	20.07	0.42	1.16	0.60	0.0616	−0.06	2.38
area (mm^2^)		≥ 48 years	18.91	0.43					
GM area/TCA	C2-C3	< 48 years	24.07	0.46	−0.64	0.66	0.3369	−2.00	0.70
ratio (%)		≥ 48 years	24.72	0.47					
TCA (mm^2^)	T9-T10	< 48 years	47.32	0.69	3.44	0.95	**0.0012**	1.49	5.39
		≥ 48 years	43.88	0.66					
Mean WM	T9-T10	< 48 years	35.30	0.63	1.85	0.84	**0.0369**	0.12	3.58
area (mm^2^)		≥ 48 years	33.45	0.55					
Mean GM	T9-T10	< 48 years	11.76	0.26	1.06	0.35	**0.0060**	0.33	1.78
area (mm^2^)		≥ 48 years	10.70	0.24					
GM area/TCA	T9-T10	< 48 years	24.83	0.52	0.44	0.72	0.5458	−1.03	1.91
ratio (%)		≥ 48 years	24.39	0.49					

PSIR measures at the C2-C3 and T9-T10 disc levels of young versus elderly persons using linear regression with gender as covariate. Mean values are least square means with adjustment for gender. Adj.: adjusted; StdErr: standard error, Diff.: difference between means, TCA: total cross-sectional area, GM: gray matter, WM: white matter. p-values are 2-sided.

### Cord measures associations with brain/vertebra metrics

The statistically significant (p < 0.05) associations between cord measures at the C2-C3 and T9-T10 levels and brain metrics extracted with the Freesurfer processing are reported in Table 4.

**Table 4:** Cord measures associations with brain metrics.

		Total cortex	Subcortical gray	Total GM	Total WM	Brain (WM+GM)
C2-C3	TCA	**0.45**	**0.72**	**0.54**	**0.62**	**0.60**
		**(0.012)**	**(<0.001)**	**(0.002)**	**(<0.001)**	**(<0.001)**
	GM	**0.44**	**0.61**	**0.50**	**0.54**	**0.54**
		**(0.015)**	**(<0.001)**	**(0.005)**	**(0.002)**	**(0.002)**
T9-T10	TCA	**0.66**	**0.71**	**0.70**	**0.63**	**0.70**
		**(<0.001)**	**(<0.001)**	**(<0.001)**	**(<0.001)**	**(<0.001)**
	GM	**0.66**	**0.79**	**0.72**	**0.63**	**0.71**
		**(<0.001)**	**(<0.001)**	**(<0.001)**	**(<0.001)**	**(<0.001)**

Pearson coefficients and respective p values (reported in brackets) for the relationships of total cross-sectional area (TCA) and gray matter (GM) area with brain tissue volumes extracted with Freesurfer, in the cohort of 32 subjects at the C2-C3 and T9-T10 disc levels. Statistically significant values are reported in bold.

**Fig 5:**
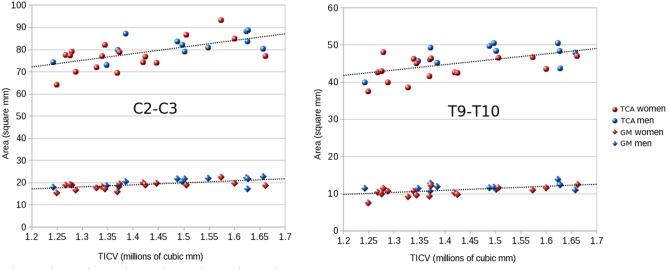
Cord measures associations with total intracranial volume. Total cross-sectional areas (TCA) and gray matter (GM) areas in function of total intracranial volume (TICV) at the C2-C3 and T9-T10 disc levels for men (blue) and women (red). Areas are reported in mm^2^, volumes in 10^6^ mm^3^. Linear regression lines for the whole cohort of subjects (cervical level: n = 32; thoracic level: n = 30) are reported.

The stronger correlations between cord measures at the C2-C3 and T9-T10 levels with TICV and other selected skull- and vertebra-derived metrics (*nasion-opistion*, *McRaes_to_C4*, *ap_vertebra_diameter*, *mean_vertebra_height*, *sagittal_vertebra_area*, *sagittal_area_trapez*) are reported in Table 5. Correlations with foramen magnum area, *McRae's line*, *ap_canal_diameter*, *middle_vertebra_height* were low and are not reported in the table. On average, the highest correlations of TCA and GM area were found with TICV (Fig. 5) and with the sagittal_vertebra_area.

**Table 5:** Cord measures associations with skull- and vertebra-derived metrics.

		TICV	sagittal_vertebra_area	sagittal_area_trapez	ap_vertebra_diameter	Mcraes_to_C4	mean_vertebra_height	nasion-inion
C2-C3	TCA	**0.63**	**0.53**	**0.42**	**0.37**	**0.45**	**0.46**	**0.45**
		**(<0.001)**	**(0.002)**	**(0.021)**	**(0.043)**	**(0.012)**	**(0.010)**	**(0.013)**
	GM	**0.62**	**0.52**	**0.50**	**0.57**	0.22	0.25	0.26
		**(<0.001)**	**(0.004)**	**(0.005)**	**(0.001)**	(0.242)	(0.178)	(0.162)
T9-T10	TCA	**0.55**	**0.52**	**0.49**	**0.43**	0.22	**0.41**	**0.48**
		**(0.002)**	**(0.005)**	**(0.009)**	**(0.021)**	(0.263)	**(0.029)**	**(0.009)**
	GM	**0.57**	**0.56**	**0.52**	**0.39**	**0.45**	**0.48**	**0.59**
		**(0.002)**	**(0.002)**	**(0.005)**	**(0.041)**	**(0.017)**	**(0.009)**	**(0.001)**

Pearson coefficients and respective p values (reported in brackets) for the relationships of total cross-sectional area (TCA) and gray matter (GM) area with skull- and vertebra-derived metrics, in the cohort of 32 subjects at the C2-C3 and 30 subjects at the T9-T10 disc levels. Out of the 11 tested metrics, only the ones with the highest correlation are reported in this table. Statistically significant values are reported in bold.

Table 6 reports the normalization coefficients obtained by linear regression with TCA and GM area as outcome variables for the models with the highest adjusted R^2^ at the C2-C3 level and the corresponding %RSD for the non-corrected measured data and the normalized values calculated with each formula.

**Table 6:** Multi-linear regression analysis of C2-C3 areas.

TCA	age (a)	TICV (b)	ap_vertebra_diameter (c)	sagittal_vertebra_area (d)	adjusted R^2^	%RSD	%RSD_meas_
Model 1	/	2.33E-05	/	0.059	0.44	5.83%	8.04%
Model 2	−0.113	2.20E-05	0.873	/	0.41	5.83%	
Model 3	−0.105	1.89E-05	/	0.063	0.47	5.56%	
**GM area**	**age (a)**	**TICV (b)**	**ap_vertebra_diameter (c)**	**sagittal_vertebra_area (d)**	**adjusted R** ^**2**^	**%RSD**	**%RSD**_**meas**_
Model 1	/	7.20E-06	/	0.017	0.42	7.41%	9.83%
Model 2a	/	7.19E-06	0.466	/	0.50	6.89%	

Normalization coefficients derived by multi-linear regression analysis with C2-C3 total cross-sectional area (TCA, top) and C2-C3 gray matter area (GM area, bottom) as outcome variables and age, total intracranial volume (TICV), ap_vertebra_diameter, sagittal_vertebra_area as independent variables (see Eq. 1 in the text). The adjusted R^2^ and the %RSD (ratios of the group standard deviation and the respective means) for the calculated values with each model are reported. %RSD_meas_ refers to the non-normalized measured data.

Mean values of the independent variables on the total cohort of subjects:

X_mean_ = Age_mean_ = 48.84 years

Y_mean_ = TICV_mean_ = 1434165.26 mm^3^

Z_mean_ = ap_vertebra_diameter_mean_ = 15.63 mm

J_mean_ = sagittal_vertebra_area_mean_ = 199.93 mm^2^

The greatest %RSD reductions were obtained for normalization with Model 3 (age, TICV, sagittal_vertebra_area) closely followed by Model 1 (TICV, sagittal_vertebra_area) and Model 2 (age, TICV, ap_vertebra_diameter) with C2-C3 TCA as outcome variable. Model 2a (TICV, ap_vertebra_diameter) was the model with the highest adj. R^2^ and %RSD reduction with C2-C3 GM area as outcome variable.

We next tested the performance of the best models found for C2-C3 (Model 3 for TCA, Model 2a for GM) at the other 3 cord levels, in terms of %RSD reduction in comparison to the measured data (Table 7).

**Table 7:** Multi-linear regression analysis at cervical and thoracic levels.

**Model 3-TCA**	**age (a)**	**TICV (b)**	**sagittal_vertebra_area (c)**	**adjusted R** ^**2**^	**%RSD**	**%RSD**_**meas**_
C3-C4	−0.150	1.17E-05	0.080	0.44	5.13%	8.41%
T8-T9	−0.101	6.58E-06	0.027	0.42	4.68%	6.95%
T9-T10	−0.110	6.36E-06	0.037	0.43	5.24%	7.78%
**Model 2-TCA**	**age (a)**	**TICV (b)**	**ap_vertebra_diameter (c)**	**adjusted R** ^**2**^	**%RSD**	**%RSD**_**meas**_
C3-C4	−0.153	1.72E-05	0.809	0.36	5.73%	8.41%
T8-T9	−0.116	6.52E-06	0.709	0.56	4.43%	6.95%
T9-T10	−0.127	6.80E-06	0.854	0.53	5.08%	7.78%
**Model 2a—GM**	**age (a)**	**TICV (b)**	**ap_vertebra_diameter (c)**	**adjusted R** ^**2**^	**%RSD**	**%RSD**_**meas**_
C3-C4	/	7.70E-06	0.172	0.37	6.36%	8.69%
T8-T9	/	1.84E-06	0.143	/	13.38%	15.49%
T9-T10	/	4.77E-06	0.191	0.33	9.31%	11.58%

Normalization coefficients derived by multi-linear regression analysis for Model 3 (age, TICV and sagittal_vertebra_area) and Model 2 (age, TICV, ap_vertebra_diameter) with total cross-sectional area (TCA) as outcome variable at the C3-C4, T8-T9 and T9-T10 disc levels.

Normalization coefficients derived by multi-linear regression analysis for Model 2a (TICV, ap_vertebra_diameter) and gray matter (GM) area as outcome variable at the C3-C4, T8-T9 and T9-T10 disc levels. The adjusted R^2^ and the %RSD (ratios of the group standard deviation and the respective means) for the calculated values are reported. %RSD_meas_ refers to the non-normalized measured data. The mean values of the independent variables for the total cohort of subjects are reported in the legend of Table 6.

In Table 7 we also report the performance of Model 2 on the TCA measures of the other 3 cord levels. This model performed even better than Model 3 at the thoracic levels. Model 2 for TCA reduced to Model 2a for GM since the variable age was no longer significant. The results suggest that the application of a single vertebral metric of simple definition (ap_vertebra_diameter) –in addition to TICV– is particularly effective for both TCA and GM normalizations in healthy controls.

## Discussions

Using a novel, reliable and clinically feasible acquisition and measurement procedure based on 2D phase-sensitive inversion recovery imaging, we explored age/gender influences on TCA, and GM and WM cord measures and assessed associations between cord measures and a) brain volumes and b) skull- and vertebra-derived metrics to develop normalization strategies to reduce variability of measures. The generally high GM/WM and WM/CSF contrast provided by all the PSIR images allowed us to segment and measure TCA and GM areas of the acquired levels, from which we derived WM areas and the GM area/TCA ratio.

The novel findings from this study include: 1) Age and gender influences on cervical spinal cord GM and WM areas with smaller areas in the elderly and women; 2) Age and gender influences on TCA, GM and WM areas at thoracic levels; 3) Strong correlations of TICV, brain WM, brain GM and total brain volume with the cervical GM area; 4) Strong correlations of TICV, brain WM, brain GM and total brain volume with the thoracic TCA and GM areas; 5) Strong correlations of vertebra-derived metrics with cervical and thoracic TCA and GM areas 6) Normalization strategies for TCA and GM areas at cervical and thoracic levels based on age, TICV, and C3 vertebral body metrics.

Some inter-subject variability of spinal cord areas has to be expected because of differences in the location of nerve roots in relation to the vertebral bodies [42]. This variability is in particular expected at the lumbar enlargement [10,11]. The T8-T9 and T9-T10 thoracic levels, above the lumbar enlargement, were chosen since we hypothesized that due to the lower inter-subject variability they could potentially offer more reliable markers of spinal cord function regarding the lower limbs. The strong correlations observed for TCA, GM and WM areas between the adjacent cervical and thoracic levels justify the choice of just one representative level per section when investigating the effect of the explored covariates.

As shown in Table 4, TCA and GM areas at the C2-C3 and T9-T10 disc levels had very strong correlations with all brain tissue volumes (total cortex, subcortical gray, total GM, total WM, total brain volumes).

With regard to the TCA at the upper cervical cord our results are in concordance with findings from Engl et al. [21], who reported strong correlations of TICV, brain WM, brain GM and total brain volume with the upper cervical TCA.

In addition to this, we now also found similar results with regard to spinal cord GM area, not only in the cervical but also in the thoracic levels of the spinal cord.

However, normalization strategies based on WM and GM tissues of healthy controls might not be appropriate in patient cohorts where the tissue used for normalization can be affected by the underlying pathology itself. We therefore also explored normalization strategies based on skull- and novel vertebra-derived metrics that are not expected to change with neurological diseases. Among the metrics we tested in this study, TICV and *sagittal_vertebra_area* showed the highest correlation coefficients with TCA and GM areas. *Sagittal_area_trapez* and *ap_vertebra_diameter* also consistently showed high correlations with TCA and GM.

A normalization formula based on TICV together with age and the *ap_vertebra_diameter* (chosen for the simplicity of its measure) reduced the %RSD of measured data at the C2-C3 cord level by 27% with regard to TCA and by 25% with regard to GM area values. The addition of a vertebral metric to age and TICV, increased the variance explained by the models at the C2-C3 level. Moreover, the application of this formula to the other cord levels also led to reductions of %RSD of TCA and GM areas (on average by 32% and 22%, respectively). This approach might, therefore, be useful to reduce anatomical driven variability of PSIR derived measures and might enhance the detection of differences between diseased and healthy subjects. However, the impact of this normalization strategy with regard to the detection of differences between patients and controls and between different disease types and degrees of disability still needs further investigation. The assumption that skull- and vertebra-derived metrics are generally not directly altered by most neurological diseases needs critical consideration on a case by case basis, taking also potentially disease associated factors into account such as side-effects of medication, increased risk of falling or consequences of immobility.

Eq. 1 reduces to formula (1) reported in Mann et al. [17] when only the TICV is used as covariate. Noticeably, the coefficients for TICV we obtained for the different models for C2-C3 TCA are very close to the coefficient reported by Mann et al., suggesting a potential for our model to be relevant to other cohorts.

The TCA, WM and GM areas showed an age-dependency with generally significantly smaller values in the elderly (at both cervical and thoracic levels), after adjustment for gender effects, with the GM area difference at C2-C3 being on the border of significance.

The measured decreases of spinal cord areas with age probably reflect age-related degenerative changes such as neuronal loss, astrocytosis, and demyelination that can be detected with MRI techniques sensitive to the microscopic characteristics of tissues. By using diffusion tensor imaging on the cervical spinal cord of healthy subjects, a decrease of fractional anisotropy with age (in particular after 40/50 years) have been reported [20,43–45]. These findings are consistent with a progressive degeneration of spinal cord tissues that could underlie the changes with age we measured at a macroscopic level.

Men generally showed larger TCA and GM areas compared to women, and a trend to larger WM areas, when adjusted for age. In the present study we did not find any age or gender effect on the GM area/TCA ratio, which is in line with similar changes of both compartments, GM and WM, in the same directions. Our observations confirm previously reported observations of age related decline of TCA in the cervical segments [18,19,21,24] and of larger cords with regard to gender differences [15,17–19,21,24], and extend the findings also to GM/WM areas and to the thoracic portion of the SC. In contrast to the majority of studies, Fradet et al. [11], did not find any age or gender influence on TCA at the cervical level, and in partial contrast to our study, significant influences of age and gender only on few of the WM and GM metrics investigated.

Limitations of the present study include the limited number of representative levels that were chosen for the analysis in the upper cervical and lower thoracic spinal cord. C2-C3 is a standard level that was already explored with regard to TCA measurements in many studies in healthy controls and MS, while the detailed TCA and GM exploration of C3-C4 and the selected thoracic levels constitutes the novel contribution of this work.

A further major limitation of the study is that the GM area was segmented manually. As both the inter- and intra-operator reliabilities were high [13,14], GM areas were delineated by a single expert in neuroradiology. A fully automated method for GM/WM segmentation was recently presented [46,47], and testing it on the contrast offered by the PSIR acquisitions would be of interest.

## Conclusions

Spinal cord TCA, GM and WM areas at the vertebral disc levels C2-C3 and T9-T10 as assessed by 2D phase-sensitive inversion recovery imaging showed age and gender dependencies. While men and younger persons generally showed larger cord areas, the ratio of GM to total cord areas did not appear to be influenced by age and gender.

Spinal cord metrics show inter-subject variablity in healthy controls. A normalization formula for TCA and GM area based on age, TICV, and C3 vertebral body metrics reduced inter-subject variability by about one third across all cord levels investigated. The applied variables can be easily measured on conventional MRI images and therefore offer a simple tool to reduce inter-subject variability.
